# Randomized, controlled trial of the long term safety, immunogenicity and efficacy of RTS,S/AS02_D_ malaria vaccine in infants living in a malaria-endemic region

**DOI:** 10.1186/1475-2875-12-11

**Published:** 2013-01-08

**Authors:** Salim Abdulla, Nahya Salim, Francisca Machera, Richard Kamata, Omar Juma, Mwanajaa Shomari, Sulende Kubhoja, Ali Mohammed, Grace Mwangoka, Thomas Aebi, Hassan Mshinda, David Schellenberg, Terrell Carter, Tonya Villafana, Marie-Claude Dubois, Amanda Leach, Marc Lievens, Johan Vekemans, Joe Cohen, W Ripley Ballou, Marcel Tanner

**Affiliations:** 1 Bagamoyo Research and Training Centre of Ifakara Health Institute, Pwani, Tanzania; 2 Muhimbili University of Health and Allied Sciences, Dar es Salaam, Tanzania; 3 Swiss Tropical and Public Health Institute, Basel, Switzerland; 4 Tanzania Commission for Science and Technology, Dar es Salaam, Tanzania; 5 London School of Hygiene and Tropical Medicine, London, UK; 6 PATH Malaria Vaccine Initiative, Washington, DC, USA; 7 GlaxoSmithKline Biologicals, Rue De L'Institut 89, Rixensart, Belgium; 8 University of Basel, Basel, Switzerland; 9 Ifakara Health Institute, 360 Kiko Avenue, Mikocheni, PO Box 78373, Dar es Salaam, Tanzania

**Keywords:** RTS,S/AS02, *Falciparum*, Malaria, Infants, Immunogenicity, Safety, Efficacy, EPI

## Abstract

**Background:**

The RTS,S/AS malaria candidate vaccine is being developed with the intent to be delivered, if approved, through the Expanded Programme on Immunization (EPI) of the World Health Organization. Safety, immunogenicity and efficacy of the RTS,S/AS02_D_ vaccine candidate when integrated into a standard EPI schedule for infants have been reported over a nine-month surveillance period. This paper describes results following 20 months of follow up.

**Methods:**

This Phase IIb, single-centre, randomized controlled trial enrolled 340 infants in Tanzania to receive three doses of RTS,S/AS02_D_ or hepatitis B vaccine at 8, 12, and 16 weeks of age. All infants also received DTPw/Hib (diphtheria and tetanus toxoids, whole-cell pertussis vaccine, conjugated *Haemophilus influenzae* type b vaccine) at the same timepoints. The study was double-blinded to month 9 and single-blinded from months 9 to 20.

**Results:**

From month 0 to 20, at least one SAE was reported in 57/170 infants who received RTS,S/AS02_D_ (33.5%; 95% confidence interval [CI]: 26.5, 41.2) and 62/170 infants who received hepatitis B vaccine (36.5%; 95% CI: 29.2, 44.2). The SAE profile was similar in both vaccine groups; none were considered to be related to vaccination. At month 20, 18 months after completion of vaccination, 71.8% of recipients of RTS,S/AS02_D_ and 3.8% of recipients of hepatitis B vaccine had seropositive titres for anti-CS antibodies; seroprotective levels of anti-HBs antibodies remained in 100% of recipients of RTS,S/AS02_D_ and 97.7% recipients of hepatitis B vaccine. Anti-HBs antibody GMTs were higher in the RTS,S/AS02_D_ group at all post-vaccination time points compared to control. According to protocol population, vaccine efficacy against multiple episodes of malaria disease was 50.7% (95% CI: -6.5 to 77.1, p = 0.072) and 26.7% (95% CI: -33.1 to 59.6, p = 0.307) over 12 and 18 months post vaccination, respectively. In the Intention to Treat population, over the 20-month follow up, vaccine efficacy against multiple episodes of malaria disease was 14.4% (95% CI: -41.9 to 48.4, p = 0.545).

**Conclusions:**

The acceptable safety profile and good tolerability of RTS,S/AS02_D_ in combination with EPI vaccines previously reported from month 0 to 9 was confirmed over a 20 month surveillance period in this infant population. Antibodies against both CS and HBsAg in the RTS,S/AS02_D_ group remained significantly higher compared to control for the study duration. Over 18 months follow up, RTS,S/AS02_D_ prevented approximately a quarter of malaria cases in the study population.

**Clinical trials:**

Gov identifier: NCT00289185

## Background

Globally, in 2010, an estimated 216 million people had malaria, 81% of which were from sub-Saharan Africa [1]. In 2010 in Africa, malaria resulted in approximately 596,000 deaths, 91% of which were in children under five years of age. Among the many approaches being pursued to control the disease, the development of safe and efficacious vaccines has been given high priority by national and international health authorities [2]. Used in conjunction with other control measures, a vaccine offers the possibility of accelerating and increasing the effectiveness of integrated malaria control.

The RTS,S/AS candidate malaria vaccine is being developed for the routine immunization of infants and children living in malaria-endemic areas as part of the Expanded Programme of Immunization (EPI). The candidate vaccine has been evaluated with two different proprietary adjuvant systems, AS02 and AS01, both indicating an acceptable safety profile in children [3-10] and infants [11-13] in clinical trials.

A Phase IIb trial was conducted between July 2006 and February 2008 in Tanzania [12]. Infants aged 6 to 10 weeks at first vaccination received either RTS,S/AS02_D_ or a hepatitis vaccine (*Engerix-B*™, GlaxoSmithKline [GSK]) as control. The RTS,S/AS02_D_ candidate malaria vaccine consists of sequences of the circumsporozoite (CS) protein and hepatitis B surface antigen (HBsAg) with the adjuvant AS02_D_ (proprietary oil-in-water emulsion formulated with MPL and QS21 immunostimulants). Both study vaccines were administered together with a vaccine containing diphtheria and tetanus toxoids, whole-cell pertussis vaccine, and conjugated *Haemophilus influenzae* type b vaccine (DTPw/Hib) (*TETRActHib*™, Aventis Pasteur).

As reported previously, over a nine-month surveillance period, serious adverse events (SAEs) were reported in a similar proportion of subjects receiving the RTS,S/AS02_D_ candidate vaccine (18.2%; 95% confidence interval [CI]: 12.7, 24.9) and hepatitis B vaccine (24.7%; 95% CI: 18.4, 31.9). Non-inferiority of the RTS,S/AS02_D_ candidate vaccine in terms of antibody responses to EPI antigens was demonstrated. RTS,S/AS02_D_ was shown to be highly immunogenic for anti-CS and -HBs antibodies. Using active detection of infection, efficacy of the RTS,S/AS02_D_ candidate vaccine against first *Plasmodium falciparum* infection was 65.2% (95% CI: 20.7, 84.7; p = 0.01) over a 6 month period [12].

This paper presents the 20 month follow up comparative data on the safety, immunogenicity and, as an exploratory endpoint, efficacy against malaria disease of RTS,S/AS02_D_ in combination with EPI vaccines in the same population of infants aged six to 10 weeks at first vaccination.

## Methods

### Study design

Details of study design, study vaccines and subject enrolment have been published elsewhere [12]. In brief, the study was a single centre, Phase IIb, randomized, controlled study conducted by the Bagamoyo Research and Training Centre, a branch of the Ifakara Health Institute (IHI; previously Ifakara Health Research and Development Centre-IHRDC) in Bagamoyo, Tanzania. The study was double-blind from months 0 to 9 and single-blind from months 9 to 20.

The protocol was approved by the Ifakara Health Institute, the National Institute of Medical Research in Tanzania, Western Institutional Review Board in the United States, the Institutional Review Board of the London School of Hygiene and Tropical Medicine, and the Swiss Tropical and Public Health Institute (Swiss TPH, previously Swiss Tropical Institute; STI) through the local government ethics committee in Basel, Switzerland. The trial was undertaken in accordance with the provisions of the International Conference on Harmonization and Good Clinical Practice guidelines and was monitored by the sponsor, GSK Biologicals, which provided both the RTS,S/AS02_D_ candidate vaccine and the hepatitis B vaccine.

The design, conduct, and results of the trial were overseen by a formally constituted Independent Data Monitoring Committee (IDMC), operating under a charter. The IDMC included experts in malaria, paediatricians, and statisticians who were appointed to oversee the ethical and safety aspects of the study conduct. The role of the IDMC included review of the implementation and progress of the study. It provided initial, regular, and closing advice on safety-related issues to the sponsor. The trial aims and procedures were explained to participating communities and written informed consent in Swahili was obtained from each child’s parent(s) or guardian(s) before study procedures were initiated. Non-literate parents or guardians indicated consent using a thumbprint, and a signature was obtained from a literate witness.

Malaria transmission in Bagamoyo area is perennial and almost entirely due to *P. falciparum.* Distribution of insecticide-treated bed nets is promoted through a National Malaria Control Programmes and artemether-lumefantrine (*Coartem*™) is currently the first-line treatment in Tanzania.

### Study subjects

Eligibility criteria included any child born to a mother who was HBsAg negative, aged between six and 10 weeks at the time of first vaccination, who did not present with any serious acute or chronic illness as determined by clinical or physical examination, medical history records or laboratory screening tests of haematology, and renal and hepatic function.

### Randomization and vaccination

Eligible subjects were randomized in a 1:1 ratio to receive three doses at 8, 12 and 16 weeks of age of either RTS,S/AS02_D_ (25 μg of lyophilized RTS,S reconstituted with 500 μL of AS02_D_ Adjuvant System) or three doses of hepatitis B vaccine (*Engerix-B*™; GlaxoSmithKline). All subjects received DTPw/Hib (*TETRActHib*™; Aventis Pasteur) at 8, 12 and 16 weeks of age.

### Surveillance of serious adverse events

A morbidity surveillance system in place at Bagamoyo District Hospital (BDH) provided a comprehensive recording of all inpatient and outpatient attendances, investigational results, diagnosis and management. All parent(s)/guardian(s) of study children were educated on the appropriate action they should take if their child became unwell at any time during the study period; they were asked not to medicate their child at home, but to seek medical care at BDH. Sick children were provided transport to go to BDH.

#### Study medical personnel

Were available 24 hours per day at BDH to receive and identify study participants when they presented, and to ensure complete investigation and documentation of the attendance. At all other dispensaries in the study area, study medical personnel were available 24 hours a day to attend to patients and facilitate the transport of study participants to BDH.

Evaluation of study subjects was according to IHI Standard Operating Procedures for the examination, investigation and documentation of each presentation. All information was recorded on an IHI clinic morbidity surveillance questionnaire. The questionnaires were subsequently reviewed by the Principal Investigator or delegate and any SAE identified as appropriate.

Verbal autopsies were to be conducted on all children who died outside a health facility to ascribe the cause of death using a questionnaire adapted from the INDEPTH standard questionnaire [14].

### Immunogenicity assessment

During the follow-up phase of the study, antibody titres for anti-CS and anti-HBs were assessed 12 months (Month 14) and 18 months (Month 20) after the third dose of vaccine. Antibody levels against CS were measured by standard ELISA methodology using plate-adsorbed R32LR antigen [NVDP(NANP)_15__2_LR [7] and expressed in ELISA Units/millilitre (EU/mL). Anti-HBs was measured using a GSK developed ELISA immunoassay, in mIU/mL. Both assays were performed at the laboratory of the Centre for Vaccinology (CEVAC), Ghent, Belgium.

### Monitoring for clinical malaria episodes

Passive case monitoring (PCD) for malaria episodes was conducted at the health facilities within the study area throughout the study period. In addition, there was monitoring for the active detection of infection (ADI) every two weeks up to the detection of a first infection or up to study month 9. In summary, two weeks before administration of the third vaccine dose, asymptomatic parasitaemia was cleared with artemether–lumefantrine comprising six doses during a three-day period. Absence of parasitaemia after treatment was confirmed by blood analysis obtained two weeks later. Any subject who continued to have parasitaemia was retreated and the event excluded from the analysis. For ADI, blood samples were taken for examination of malaria parasitaemia at each visit. For PCD, any study subject presenting with fever (axillary temperature ≥37.5°C) within the preceding 24 hours underwent a blood draw for the determination of malaria parasites. Severe malaria was defined prospectively according to an agreed case definition and confirmed by medical review (Table 1).

**Table 1:** Severe malaria definitions for reporting of SAEs

Severe malaria anaemia	Asexual *P. falciparum* parasitemia > 0 definitive reading
	Haematocrit < 15%^1^
	No other more probable cause of illness
Cerebral malaria	Asexual *P. falciparum* parasitemia > 0 definitive reading
	Coma score ≤ 2^2^
	No other identifiable cause of loss of consciousness
Severe malaria (other)	Asexual *P. falciparum* parasitemia > 0 definitive reading
	No other more probable cause of illness
	Does not meet criteria for severe malaria anaemia or cerebral malaria
	One of the following:
	Multiple seizures^3^
	Prostration^4^
	Hypoglycaemia^5^
	Acidosis
	Circulatory collapse

### Statistical analysis

An analysis plan was prospectively agreed upon by the IDMC, sponsor and investigators. Statistical analyses were conducted using SAS version 8 (SAS, Cary, NC, USA). The primary study endpoint was of safety (occurrence of SAEs up to Month 20) with secondary endpoints of immunogenicity (anti-CS and anti-HBs antibody titres to Month 20). Additional endpoints for efficacy were also explored. All subjects who had received at least one dose of study vaccine were included in the safety analysis. The proportion of subjects with a SAE, classified by the Medical dictionary for regulatory activities (MedDRA) preferred term level [15], was tabulated with exact 95% CI. Episodes of severe malaria were tabulated with exact 95% Confidence Intervals (CI).

The primary analysis for immunogenicity was performed on the According to Protocol (ATP) cohort, which included all evaluable subjects (i.e. those meeting all eligibility criteria, complying with the procedures defined in the protocol, with no elimination criteria during the study) and for whom data concerning immunogenicity endpoints were available. The percentage of subjects with seropositive levels of anti-CS antibodies (cut-off ≥ 0.5 EU/mL) and seroprotective levels of anti-HBs antibodies (cut-off ≥ 10 mIU/mL) with 95% CI were assessed. Antibody titres were summarized by geometric mean titres (GMT) with 95% CI for both antigens.

The primary exploratory analysis for efficacy was performed on the ATP cohort, which included all enrolled subjects for whom data concerning efficacy endpoint measures were available, who received all 3 doses of study vaccine according to the randomization list, received clearance drug and were parasite negative at the start of the ADI period. The study evaluated efficacy against clinical malaria, according to primary and secondary case definitions (see Table 2). Time at risk started 14 days post dose 3 and was corrected for absences from the study area and anti-malarial drug use. Vaccine efficacy (VE) against first or only episodes of clinical malaria was assessed using Cox regression models, defined as 1-R where R is the hazard ratio of the RTS,S/AS02_D_ group against control (with 95% CI). Schoenfeld p-values and models with time varying covariates were evaluated to check proportionality of hazards. VE against multiple episodes was calculated using Poisson regression with random effects defined as 1- incident rate ratio. For the ATP analyses, VE estimates were adjusted for village of residence and distance to BDH.

**Table 2:** Case definitions for clinical malaria

**Primary Case Definition**	· The presence of *P. falciparum* asexual parasitaemia above 500 per μL) on Giemsa stained thick blood films AND
	· the presence of fever (axillary temperature ≥ 37.5°C)
**Secondary Case Definition**	· The presence of *P. falciparum* asexual parasitaemia above 0 per μL) on Giemsa stained thick blood films AND
	· the presence of fever (axillary temperature ≥ 37.5°C) or history of fever in the previous 24 hours

## Results

### Subject cohort

**Figure 1:**
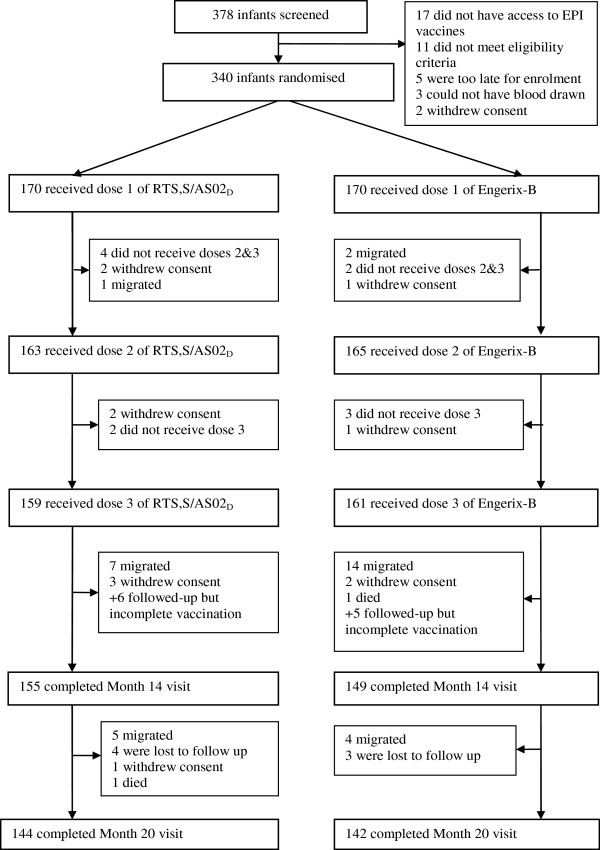
**CONSORT diagram for study participants.**^a^ Subjects were temporarily out of study area but returned for the Month 14 visit. ^b^ Other: investigator decided not to continue vaccination as EPI vaccination documentation was not available, this subject returned for the Month 14 visit. ^c^ One subject died after consent withdrawal; death was not considered related to vaccination.

A total of 340 infants were randomly assigned to a study group and received at least one vaccination (Total Vaccinated Cohort: 170 RTS,S/AS02_D_, 170 hepatitis B vaccine). Figure 1 summarizes subject participation during the course of the study. The month 20 visit was completed by 144 subjects in the RTS,S/AS02_D_ group and 142 subjects in the hepatitis B vaccine group. From month 0 to 20, 54 subjects withdrew from the study, 26 from the RTS,S/AS02_D_ group and 28 from the hepatitis B group. The main reason for withdrawal in both groups was migration out of the study area.

The basic parameters of those withdrawing were not different compared to the remaining study population. The demographic profile of subjects in the RTS,S/AS02_D_ and hepatitis B vaccine groups was balanced in terms of age and gender. At the time of first vaccine administration the mean age of subjects was 7.8 (SD 0.77) weeks and 7.9 (SD 0.83) weeks in the RTS,S/AS02_D_ and hepatitis B vaccine groups, respectively. A similar proportion of subjects were males in both the RTS,S/AS02_D_ and hepatitis B vaccine groups (47% and 50%, respectively).

### Safety outcomes

From the time of first vaccination until Month 20, 57 (33.5%) recipients of RTS,S/AS02_D_ and 62 (36.5%) recipients of hepatitis B vaccine, in co-administration with DTPw/Hib, reported at least one SAE (Table 3). No SAE was considered by the investigator to be related to vaccine. There were no concerning imbalances in the cause of SAEs.

**Table 3:** SAEs occurring in more than one subject classified by MedDRA preferred term (Total Vaccinated Cohort [Months 0–20])

	**Hepatitis B vaccine N = 170**				**RTS,S/AS02**_**D**_**N = 170**			
**Preferred Term**	**n**	**%**	**95% CI**		**n**	**%**	**95% CI**	
At least one SAE	62	36.5	29.2	44.2	57	33.5	26.5	41.2
Pneumonia	36	21.2	15.3	28.1	25	14.7	9.7	20.9
*P. falciparum* infection	25	14.7	9.7	20.9	19	11.2	6.9	16.9
Anaemia	16	9.4	5.5	14.8	16	9.4	5.5	14.8
Gastroenteritis	12	7.1	3.7	12.0	16	9.4	5.5	14.8
Febrile convulsion	2	1.2	0.1	4.2	5	2.9	1.0	6.7
Urinary tract infection	3	1.8	0.4	5.1	4	2.4	0.6	5.9
Lymphadenitis	0	0.0	0.0	2.1	3	1.8	0.4	5.1
Abscess	2	1.2	0.1	4.2	2	1.2	0.1	4.2
Bronchiolitis	1	0.6	0.0	3.2	2	1.2	0.1	4.2
Sepsis	2	1.2	0.1	4.2	2	1.2	0.1	4.2
Upper respiratory tract infection	3	1.8	0.4	5.1	2	1.2	0.1	4.2
Convulsion	2	1.2	0.1	4.2	2	1.2	0.1	4.2
Acarodermatitis	2	1.2	0.1	4.2	0	0.0	0.0	2.1
Bronchial hyperreactivity	3	1.8	0.4	5.1	0	0.0	0.0	2.1

At least one symptom = at least one symptom experienced (regardless of the MedDRA Preferred Term).

N = number of subjects with at least one administered dose.

n/% = number/percentage of subjects reporting at least once the symptom.

95% CI = exact 95% confidence interval.

Two SAEs were fatal, one occurring during the double-blind phase (severe pneumonia/symptomatic seizure) in a subject from the hepatitis B vaccine group and one during the single-blind phase of the study (cerebral malaria) in a subject from the RTS,S/AS02_D_ group. On analysis of predefined cases of severe malaria, five subjects in the RTS,S/AS02_D_ group and four subjects in the hepatitis B vaccine group were reported as having severe malaria. Apart from the fatal SAEs, no subject was withdrawn from the study due to an adverse event.

### Immunogenicity outcomes

#### Anti-CS response

Pre-vaccination, a similar proportion of subjects was seropositive for anti-CS antibodies in the RTS,S/AS02_D_ and hepatitis B vaccine, co-administered with DTPw/Hib, groups (23.4% and 25.7%, respectively) (Table 4). The pre-vaccination anti-CS antibody GMTs were below the assay cut-off (<0.5 EU/mL) in both groups.

**Table 4:** Seropositivity rates and GMTs for anti-CS antibodies from Months 0 to 20 (ATP Cohort for Immunogenicity)

**Group**	**Timing**	**N**	**Seropositive**				**GMT (EU/mL)**		
			**n**	**%**	**95% CI**		**value**	**95% CI**	
RTS,S/AS02_D_	SCREENING	141	33	23.4	16.7	31.3	0.3	0.3	0.4
	Post Dose 2 (Month 2)	151	149	98.7	95.3	99.8	28.9	22.4	37.3
	Post Dose 3 (Month 3)	143	141	98.6	95.0	99.8	69.5	53.9	89.6
	Post Dose 3 (Month 9)	143	127	88.8	82.5	93.5	6.2	4.6	8.3
	Post Dose 3 (Month 14)	142	107	75.4	67.4	82.2	3.0	2.2	4.0
	Post Dose 3 (Month 20)	131	94	71.8	63.2	79.3	1.9	1.4	2.6
Hepatitis B	SCREENING	152	39	25.7	18.9	33.4	0.4	0.3	0.4
vaccine	Post Dose 2 (Month 2)	156	10	6.4	3.1	11.5	0.3	0.3	0.3
	Post Dose 3 (Month 3)	144	2	1.4	0.2	4.9	0.3	0.2	0.3
	Post Dose 3 (Month 9)	147	0	0.0	0.0	2.5	0.3	0.3	0.3
	Post Dose 3 (Month 14)	139	2	1.4	0.2	5.1	0.3	0.2	0.3
	Post Dose 3 (Month 20)	132	5	3.8	1.2	8.6	0.3	0.3	0.3

Seropositive ≥ 0.5 EU/mL.

N = number of subjects with available results.

n/% = number/percentage of subjects with titre within the specified range.

At month 14 and month 20, 75.4% and 71.8% of subjects in the RTS,S/AS02_D_ group were seropositive for anti-CS antibodies, compared to 1.4% and 3.8% of subjects in the hepatitis B vaccine group. At month 14, anti-CS antibody GMTs in the RTS,S/AS02_D_ were low (3.0 EU/mL) which decreased further at month 20 (1.9 EU/mL), but were still higher than the hepatitis B control group (<0.5 EU/mL).

#### Anti-HBs response

Subjects enrolled in this study were born to HBsAg-negative mothers and no neonatal Hepatitis B immunization programme was in place. Thus, the subjects had received no previous Hepatitis B vaccine. Prior to vaccination, the proportion of subjects with seroprotective levels of maternal anti-HBs antibodies derived from passive transfer during pregnancy was similar in the RTS,S/AS02_D_ and hepatitis B vaccine, co-administered with DTPw/Hib, groups (38.3% and 34.4%, respectively) (Table 5). On average, pre-vaccination anti-HBs antibody GMTs were low (<15 mIU/mL).

**Table 5:** Seroprotective rates and GMTs for anti-HBs antibodies from Months 0 to 20 (ATP Cohort for Immunogenicity)

**Group**	**Timing**	**N**	**Seroprotected**				**GMT (mIU/mL)**		
			**n**	**%**	**95% CI**		**Value**	**95% CI**	
RTS,S/AS02_D_	SCREENING	115	44	38.3	29.4	47.8	14.5	10.9	19.2
	Post Dose 2 (Month 2)	148	140	94.6	89.6	97.6	110.9	89.1	138.1
	Post Dose 3 (Month 3)	140	140	100	97.4	100	667.3	532.9	835.7
	Post Dose 3 (Month 20)	131	131	100	97.2	100	1520.6	1206.8	1915.8
Hepatitis B	SCREENING	131	45	34.4	26.3	43.1	13.3	10.0	17.6
vaccine	Post Dose 2 (Month 2)	147	82	55.8	47.4	64.0	17.1	13.6	21.4
	Post Dose 3 (Month 3)	141	133	94.3	89.1	97.5	113.8	91.3	141.8
	Post Dose 3 (Month 20)	132	129	97.7	93.5	99.5	184.3	144.4	235.4

Seroprotected ≥ 10 mIU/mL.

N = number of subjects with available results.

n/% = number/percentage of subjects with titre within the specified range.

Seroprotective levels of anti-HBs antibodies at month 20 were achieved in 100% of recipients of RTS,S/AS02_D_ co-administered with DTPw/Hib and 97.7% of recipients of hepatitis B vaccine co-administered with DTPw/Hib. The anti-HBs antibody GMT at Month 20 was greater in recipients of RTS,S/AS02_D_ (1521 mIU/mL) than in recipients of hepatitis B vaccine (184 mIU/mL).

### Efficacy outcomes

**Figure 2:**
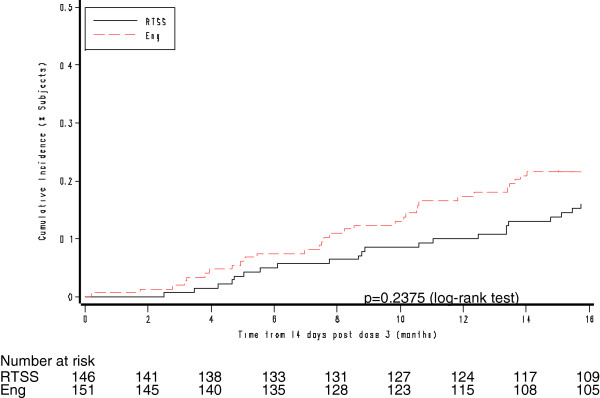
**Kaplan-Meier survival curves showing the cumulative incidence of *P. falciparum *disease, Primary Case Definition (ATP Cohort for Efficacy [Months 2.5 - 20]).** Eng = Engerix-B Hepatitis B vaccine.

Over 12-month and 18-month periods following Dose 3 of study vaccine, VE of RTS,S/AS02_D_ against the primary case definition for multiple episodes of disease was 50.7% (p = 0.072) and 26.7% (p = 0.307), respectively, and for first or only episode of clinical malaria 53.6% (p = 0.026) and 34.9% (p = 0.101), respectively (Table 6). Figure 2 shows Kaplan–Meier curves of the cumulative incidence of first or only malaria episodes in the two study groups (Primary Case Definition). In the intention to treat (ITT) population vaccine efficacy against all episodes of malaria over the entire follow up starting at dose 1 was 14.4% (95% CI: -41.9 to 48.4, p = 0.545).

**Table 6:** **Vaccine efficacy against *P. falciparum *disease (ATP Cohort for Efficacy)**

**RTS,S/AS02**_**D**_			**Hepatitis B Vaccine**			**Point estimate of VE adjusted for covariates**			
**Subjects (N)**	**No. of events**	**PYAR**	**Subjects (N)**	**No. of events**	**PYAR**	**(%)**	**95% CI**		**P value**
**Primary Case Definition: multiple episodes**									
*Months 2.5 – 14*									
146	18	124.48	151	30	127.91	50.7	−6.5	77.1	0.072
*Months 2.5 – 20*									
146	42	193.41	151	51	198.29	26.7	−33.1	59.6	0.307
**Primary Case Definition: first or only**									
*Months 2.5 – 14*									
146	13	121.04	151	24	121.34	53.6	8.6	76.4	0.026
*Months 2.5 – 20*									
146	26	181.92	151	34	178.56	34.9	−8.8	61.1	0.101

*PYAR*: Episodes/Person Years at Risk; *VE*: Vaccine Efficacy (1-HR); *CI*: Confidence Interval; p value from Cox PH model; Poisson regression for multiple episodes.

Over the same time periods, respectively, VE of RTS,S/AS02_D_ against the Secondary Case Definition for multiple episodes of disease was 53.5% (95% CI: 3.3, 77.6; p = 0.040) and 26.2% (95% CI: -19.0, 54.2; p = 0.212) and for first or only episode of clinical malaria was 55.7% (95% CI: 15.4, 76.8; p = 0.014) and 32.0% (95% CI: -6.4, 56.5; p = 0.091). A test based on the Schoenfeld residuals (p = 0.1919) suggested no violation of the proportional hazards assumption over the follow up period, consistent with a constant effect over the course of the study.

## Discussion

This paper further contributes key information on the safety, immunogenicity and efficacy profile over a 20 month follow up period of the candidate malaria RTS,S adjuvanted vaccine when co-administered to infants alongside routine immunizations included in the Expanded Programme of Immunization.

This study shows that, over 12 and 18 months of follow up post vaccination: vaccine efficacy against multiple episodes of malaria disease was 50.7% and 26.7%, respectively, in the ATP population. Vaccine efficacy against multiple episodes of malaria disease in the ITT population, starting at first dose of vaccine, was lower (14%). Including ITT analysis provides fair comparisons among the vaccinated population, but perfect adherence to the protocol, especially for phase II studies, is crucial. Results from ATP analyses aim to predict the true biological efficacy of the vaccine.

Initial results demonstrated that RTS,S/AS02_D_ has an acceptable safety profile, that it can be given in co-administration with EPI vaccines and provides protection against first *P. falciparum* infection of approximately 65% (p = 0.01) over 6 months of follow up [12].

As an exploratory endpoint in this follow up study, vaccine efficacy against multiple episodes of clinical disease was 51%, though not achieving statistical significance (p = 0.072), and 54% against first or only episode of clinical disease (p = 0.026), over 12 months post-vaccination. These results are consistent with those of a trial evaluating safety, immunogenicity and efficacy of RTS,S/AS01 in co-administration with EPI vaccines in infants [13]. Similar levels of protection have been observed in children 5–17 months old upon first RTS,S/AS01 vaccination in a Phase II trial conducted in Tanzania and Kenya [16].

However, the large multi-country, multi-site RTS,S/AS01 Phase III trial showed that in young infants the vaccine provided modest protection against malaria when co-administered with EPI vaccines [17]. The fact that most of the participants in the Phase III study come from high transmission areas indicates that RTS,S protection may be influenced by other factors including level of transmission.

While CIs are wide, and models, including time-varying covariates and Schoenfeld residuals (p = 0.192), do not support waning in vaccine efficacy, a longer follow-up of infants post-vaccination showed lower levels of protection in this trial (27% over 18 months), which was not significant at the 5% level (p = 0.307). This is in contrast with a study with the RTS,S/AS01 formulation in infants, which showed protection of 59% over 19 months of follow up (p < 0.001) [18].

The reduction in the level of protection observed after 18 months, when compared to the level observed at 12 months, was also reported in a study with 45 months of follow up [19]. While it is not impossible that the close follow up during the ADI period may have impacted VE estimates in this trial, a possible explanation could be due to a combination of (i) true decay in vaccine-induced protection over the period of follow up, (ii) the decline in the number of susceptible subjects due to rapid acquisition of immunity, and/or (iii) variability in malaria transmission and exposure. Similar observations were made in recent long-term follow-up studies of RTS,S/AS02 [6,7,9-12,20,21].

The assessment of anti-CS antibody titres shows that at 18 months post vaccination 71.8% of RTS,S/AS02 recipients remain seropositive. While remaining higher than those seen in the control group, antibody levels are much lower than early after vaccination. Although no protective threshold has been established, anti-CS antibody levels have been shown to be associated with protection [18,22,23]. The relatively low immune responses of RTS,S/AS02 to anti-CS observed in this trial, compared to the AS01_E_ formulation, may have also contributed to the apparent drop in long-term protection.

As seen in other studies of RTS,S/AS02 and RTS,S/AS01 [3-13,22,23], post vaccination anti-HBs seroprotection rates and GMTs are high. In this study, at month 20 they remained greater than in recipients of hepatitis B vaccine. This supports the fact that the RTS,S candidate vaccine also confers protection against Hepatitis B virus (HBV).

The observation of an acceptable safety profile of RTS,S/ASO2_D_ over 20 months of follow up is consistent with long term safety follow up of RTS,S/AS01 in infants [13]. No safety concerns appeared upon SAE review over the duration of follow up. Few children died and severe malaria rates were lower than expected in this setting, which may be due to close follow up of this cohort. Clinically diagnosed pneumonia, reported as SAEs and classified by MedDRA preferred terms [15], tended to be more frequently reported among hospitalized participants in the hepatitis B vaccine group at 9 months post vaccination [12], and this effect was less marked in the current data set to 20 months. A tendency for pneumonia rates to be lower may be due to a variety of reasons, including chance findings, lack of accurate diagnosis for pneumonia and/or the possibility that the malaria candidate vaccine reduced the indirect consequences of malaria. A more rigorous assessment of the co-morbidities is ongoing in the Phase III trial [17,22].

The acceptable vaccine safety profile and the efficacy data obtained in this trial in the context of co-administration of EPI vaccines adds to the growing body of evidence that, if approved, the RTS,S candidate vaccine could contribute to the reduction of the malaria disease burden in infants and children and become an additional component of integrated malaria control strategies.

### Trademarks

*Coartem*™ is a registered trademark of Novartis Pharma AG.

*Engerix*™ is a registered trademark of GlaxoSmithKline.

*TETRActHib*™ is a registered trademark of Aventis Pasteur.

GSK Study ID number: 104298 (Malaria-040).

## Competing interests

This study was sponsored by GSK Biologicals SA Belgium, and funded by both GSK Biologicals and the PATH Malaria Vaccine Initiative (MVI). MVI supports the development and testing of several malaria vaccine candidates. MAD, MCD, AL, ML, JV, JC, and WRB are employees of the GlaxoSmithKline group of companies. MAD, MCD, AL, JV, JC and WRB own shares in GSK Biologicals. JC and WRB are listed as the inventors of patented malaria vaccines. However, neither individual holds a patent for a malaria vaccine. TC and TV were, at the time of the study, employees at PATH MVI. DS declares his institution receiving consultancy fees from MVI for other work. MT is a board member of the UBS-Optimus foundation and declares his institution received compensation for his membership of a Novartis scientific advisory board, and reimbursements from the Bill & Melinda Gates Foundation and Wellcome Trust as compensation for travel costs. None of the other authors declare any further potential competing interests.

## Authors’ contributions

All authors contributed to the design, implementation, analysis, and interpretation of the study. SA was the principal investigator for the trial and directed the malaria vaccine teams in Bagamoyo. MT assisted SA as co-investigator and site partner. RO, SA, TA and AU guided the implementation team. SA was implicated in all phases of the study and led the write up of the manuscript, which all other authors contributed to. AL, JV, RB and JC led the research, clinical and safety activities at GSK. ML and AL led the data analysis. RO, OJ, FM, MS, AU and TA were profoundly implicated in field and hospital activities, and safety surveillance. Marie-Claude Dubois from GSK and OJ from IHI were the malaria vaccine project leaders. MAD coordinated the immunology read-out team. CM supervised all laboratory work at BRTC. AL, JV, ML, RB and JC from GSK contributed to the design, implementation, and interpretation of this trial and the malaria vaccine programme at GSK. MT provided key support through the trial. TV was Director at the PATH Malaria Vaccine Initiative. All authors read and approved the final manuscript.
